# Atypical Miller Fisher Syndrome with Anisocoria and Rapidly Fluctuating Pupillary Diameter

**DOI:** 10.1155/2015/472843

**Published:** 2015-08-24

**Authors:** Garima Gupta, Antonio Liu

**Affiliations:** ^1^Department of Internal Medicine, White Memorial Medical Center, Loma Linda University School of Medicine, 1720 E. Cesar Chavez Avenue, Los Angeles, CA 90033, USA; ^2^Department of Neurology, White Memorial Medical Center, Loma Linda University School of Medicine, 1720 E. Cesar Chavez Avenue, Los Angeles, CA 90033, USA

## Abstract

Miller Fisher syndrome is a variant of Guillain-Barre syndrome characterized by the classic triad of ophthalmoplegia, ataxia, and areflexia. Pupillary involvement is common in MFS and has been reported in 35–42% of MFS patients. Although case reports have discussed isolated ophthalmoplegia as a presentation of MFS, anisocoria and rapid fluctuation of pupillary diameter have not been reported in anti-GQ1b antibody positive individuals. Here we describe an individual who presented with diplopia and was found to have progressive internal and external ophthalmoplegia with frequent fluctuations in pupillary diameter and anisocoria. These exam findings are not commonly described even in atypical presentations of MFS. The onset of symptoms was preceded by an upper respiratory infection but no gastrointestinal symptoms. Imaging and CSF studies were unremarkable; however serum levels of immunoglobulin G anti-GQ1b antibody and anti-GAD antibody were elevated confirming the diagnosis of MFS. The patient was treated with IVIG and intravenous steroids with mild resolution of external ophthalmoplegia. He did not go on to develop more typical features of MFS such as ataxia or areflexia. This demonstrates that isolated external and internal ophthalmoparesis with rapidly fluctuating pupillary diameter and associated anisocoria can be the sole manifestation of atypical MFS.

## 1. Introduction

Miller Fisher syndrome is a variant of Guillain-Barre syndrome characterized by the classic triad of ophthalmoplegia, ataxia, and areflexia [1]. It has been reported that more than 90% of patients with acute MFS have IgG antibodies against GQ1b [2]. Pupillary dysfunction has been reported in 35–42% of MFS patients [3]. Several case reports discuss atypical presentations of MFS with isolated ophthalmoplegia or pupillary dysfunction [3–5]. However, there have been no case reports of patients presenting with isolated ophthalmoplegia and anisocoria with frequent fluctuations in pupillary diameter. We describe a patient with atypical MFS confirmed with serum anti-GQ1b antibodies and anti-GAD antibodies presenting with progressive external and internal ophthalmoplegia, anisocoria, and rapid, frequent, and wide fluctuations in pupillary diameter.

## 2. Case Report

This patient is a 59-year-old Hispanic male with twelve-day headache, four-day diplopia, and ptosis of the left eye. He described a mild upper respiratory infection one month prior to admission. He denied any ataxia. On initial exam the patient had ptosis of both eyes and complete ophthalmoplegia. He was unable to look up, down, right, or left. He also had anisocoria with a pinpoint pupil on the right and a six-millimeter blown pupil on the left. Neurological exam was otherwise unremarkable including symmetrical and intact deep tendon reflexes in all extremities, normal gait, and no cerebellar dysfunction. Just hours later the patient was found to have pinpoint pupils bilaterally, and later in the day he had dilation to three mm bilaterally. Subsequent ophthalmological exam including mydriatics confirmed complete ophthalmoplegia. Fundoscopic exam revealed normal pressure in both eyes and normal appearing fundus and retina. On day two of admission the pupils remained at three mm bilaterally. On day three the pupils became nonreactive bilaterally but remained three mm in diameter. On day four of admission the pupils became further dilated to six mm bilaterally and continued to be nonreactive. The pupils remained dilated with complete ophthalmoplegia, and the patient did not develop typical features of MFS such as ataxia or areflexia.

MRI and MRA of the brain and orbits were unremarkable. Acetylcholine receptor antibodies were also negative. Oral pyridostigmine did not have any effect on pupillary diameter. A lumbar puncture showed normal opening pressure and elevated protein of 124 mg/dL. CSF myelin basic protein was negative. Methylprednisolone 250 mg IV every six hours and intravenous immunoglobulin 35 gm daily were started given a high index of suspicion for MFS. Serum anti-GQ1b antibodies IgG and IgM returned elevated at 373 IU on day four of IVIG therapy. Other antibodies GD1b, GD1a, GM1, and GM2 were all negative. After discharge an anti-GAD antibody returned elevated at 142 IU/mL. The patient received five-day IVIG with mild improvement in extraocular movements, continued pupillary dilation, and resolution of diplopia.

## 3. Discussion

Miller Fisher syndrome is a variant of Guillain-Barre syndrome. The incidence of GBS is about one to two in 100,000 and MFS is only one to seven percent of such cases [6]. Ophthalmoparesis and diplopia are considered early findings of MFS, and pupillary abnormalities indicating internal ophthalmoplegia are well described. Our patient presented with isolated internal and external ophthalmoplegia without other typical features of MFS. In addition, he exhibited anisocoria and rapid fluctuations in pupillary size. These findings have not been commonly described in an anti-GQ1b antibody positive individual.

Two recent case reports discuss pupillary abnormalities in MFS. Park and Lee [3] describe a patient with isolated internal ophthalmoplegia as the sole manifestation of MFS after infection with Epstein-Barr virus. The patient had no evidence of external ophthalmoplegia or ataxia, but her pupils were anisocoric with mydriasis and sluggish response to light. Unlike our patient there is no discussion of changes in pupillary diameter. In addition, serum anti-GQ1b antibodies were negative. MFS was diagnosed based on CSF albuminocytologic dissociation. This patient was treated with five-day IVIG. Bae et al. [4] describe a patient with bilateral mydriasis and dilation of pupils indicative of internal ophthalmoplegia as an isolated finding of MFS without external ophthalmoparesis or ataxia. This patient went on to develop typical features of MFS and anti-GQ1b antibodies were positive. Unlike our patient, anisocoria was not present in this case.

Pupillary involvement and mydriasis can be seen in 50% of patients with MFS [4]. Recent literature shows that the index of suspicion must be high for MFS as patients may not present with the classic triad of ataxia, areflexia, and ophthalmoplegia. Up to 18% of patients may have intact reflexes [6]. Diagnosis is made with detection of elevated anti-GQ1b antibodies. Another report suggests that serum and CSF anti-GAD antibodies may also be associated with MFS [2]. Our case highlights that variations in pupillary involvement may be present in patients with MFS that do not present with the typical features. Our patient did have ophthalmoparesis and diplopia; however he did not go on to develop ataxia or areflexia. Internal and external ophthalmoplegia were present and anti-GQ1b antibodies and anti-GAD antibodies returned positive. The most striking feature was the development of anisocoria and rapid, frequent, and wide fluctuation in pupillary diameter during the course of a single day. Our case shows that isolated external and internal ophthalmoparesis with variations in pupillary diameter and associated anisocoria can be the sole manifestation of atypical MFS.
